# Immunosenescence and its impact on ischemic stroke risk and outcomes in older adults: a systematic review

**DOI:** 10.3389/fnagi.2026.1776458

**Published:** 2026-03-09

**Authors:** Celest Wen Ting Seah, Matthias Ho, Collin Chu, Karishma Sachaphibulkij, Paul A. MacAry, Laura McCulloch, Velda Xinying Han, Benjamin Yong-Qiang Tan, Vanda Wen Teng Ho

**Affiliations:** 1 Department of Medicine, Yong Loo Lin School of Medicine, National University of Singapore, Singapore, Singapore; 2 Life Sciences Institute, National University of Singapore, Singapore, Singapore; 3 Centre for Inflammation Research, Institute for Regeneration and Repair, Edinburgh, United Kingdom; 4 Division of Paediatric Neurology, Department of Paediatrics, Khoo Teck Puat – National University Children's Medical Institute, National University Hospital, Singapore, Singapore; 5 Department of Paediatrics, National University of Singapore, Singapore, Singapore; 6 Division of Neurology, Department of Medicine, National University Hospital, Singapore, Singapore; 7 Division of Geriatric Medicine, Department of Medicine, National University Hospital, Singapore, Singapore

**Keywords:** immunology, immunosenescence, inflammaging, ischemic stroke, neurology, systemic review

## Abstract

**Background:**

Age is a major risk factor for ischemic stroke (IS), with immunosenescence–age-related immune system dysfunction - contributing to worse outcomes. Immunosenescence impairs immune responses, heightens inflammation, and increases susceptibility to infections, all of which affect stroke prognosis. This review investigates the association between immunosenescence, immune cell dysfunction, and IS risk and outcomes.

**Methods:**

A systematic review was conducted to identify cohort studies examining immunosenescence in IS patients aged 60 and above. Databases PubMed and Embase were searched up to 10 August 2024. Studies were included if they analyzed immune cell markers or inflammatory markers in relation to IS risk or outcomes. A total of 11 studies met the inclusion criteria.

**Results:**

Elevated inflammatory markers such as interleukin (IL)-6, high-sensitivity C-reactive protein (hs-CRP), and Th17 cells were significantly associated with poorer stroke outcomes. Studies indicated an imbalance between pro-inflammatory Th17 cells and regulatory T cells (Treg) post-stroke. Higher neutrophil-to-lymphocyte ratio (NLR) and alterations in B-cell subsets were also observed in older stroke patients, further contributing to the inflammatory response. These immune dysregulations were linked to increased mortality and poor recovery.

**Conclusion:**

Immunosenescence plays a crucial role in IS pathogenesis and recovery, with chronic inflammation and immune dysfunction exacerbating stroke outcomes in older adults. Targeting immune markers, particularly IL-6 and the Th17/Treg imbalance, may offer new therapeutic approaches to improve stroke prognosis in aging populations. Further research is needed to develop interventions that address immunosenescence in IS.

**Systematic review registration:**

https://www.crd.york.ac.uk/PROSPERO/view/CRD42024583142.

## Introduction

Age is a key determinant of ischemic stroke (IS) risk and outcomes (Drozdowska et al., 2019; Sykes et al., 2021). A deeper understanding of the biological changes associated with aging may uncover new therapeutic targets for IS. In humans, it is increasingly recognized that the immunity declines with age. Immune cells display diminished functional capacity for antigen presentation, cell trafficking, and responses to cytokine stimulation (Selvaraj et al., 2016). The system becomes immunosenescent, producing immune responses with reduced specificity and effectiveness to antigenic stimulation (Deleidi et al., 2015; Ventura et al., 2017; Goronzy and Weyand, 2003). This is linked with a higher incidence of infections, neoplasia, and autoimmune diseases (Drozdowska et al., 2019; Pawelec et al., 2006; Hakim et al., 2004). Additionally, there is an age-related increase in inflammatory markers, such as C-reactive protein (CRP), interleukin-6 (IL-6), and tumor necrosis factor (TNF), which are associated with worse stroke outcomes, increased pneumonia risk, and higher mortality (Bruunsgaard et al., 1999; Van Den Biggelaar et al., 2004; Libby et al., 2010).

IS is a leading cause of mortality and disability, conveying a substantial socioeconomic burden on both healthcare systems and individuals, particularly in countries with aging populations and advanced healthcare infrastructure (Collaborators GBDS, 2021; Feigin et al., 2025). Despite advancements in treatments such as thrombolytic therapy and endovascular thrombectomy that have decreased mortality rates, the persistent neural damage resulting from ischemia–reperfusion remains a significant challenge that often leads to multi-organ dysfunction. In this complex pathophysiological cascade, dysfunction in the immune system is strongly implicated, particularly characterized by systemic inflammatory reactions. Neuroinflammation, initiated in the aftermath of IS, plays a pivotal role in these immune processes, significantly contributing to the overall pathological sequelae (Wu et al., 2022). The first 24 h after an ischemic stroke are marked by a profound ‘cytokine storm’, where proinflammatory mediators and chemokines flood the systemic circulation. This hyper-acute phase triggers an immediate mobilization of innate immune cells, reshaping the peripheral immune environment. By altering this landscape rapidly, the body effectively primes itself for the wave of neuroinflammation and the various clinical complications that often follow in the wake of the initial event (Wu et al., 2022; Kowalski et al., 2023). In individuals with an aging immune system, the ability to maintain a controlled inflammatory response is impaired, leading to chronic, uncontrolled inflammation and an increased recruitment of peripheral immune cells. This dysregulated immune response is associated with higher levels of pro-inflammatory cells, which are positively correlated with worse post-stroke outcomes and a higher risk of post-stroke infections (Finger et al., 2022; Gallizioli et al., 2023).

Immunosenescence is not merely a decline in immune activity but a profound systemic remodeling. At its core lies ‘inflammaging’ – a persistent, low-grade inflammatory state driven by the accumulation of senescent cells and their pro-inflammatory secretory profiles. This shift reconfigures the cerebral vasculature, priming it for injury. During an ischemic stroke, this pre-existing dysregulation actively dictates the severity of neural damage and stymies the resolution of inflammation. While prognosis has traditionally been tied to chronological age, mounting evidence suggests this underlying immune dysfunction is what directly exacerbates tissue loss and restricts neuroplasticity in older adults (Gallizioli et al., 2023; Swardfager et al., 2014). In the aging immune system, the depletion of naive T-cells is accompanied by a problematic build-up of senescent, pro-inflammatory memory cells. This shift creates a volatile environment that primes the brain for more severe damage during an ischemic event. Moreover, this dysregulated state acts as a barrier to recovery, stymying the essential transition from acute inflammation to the repair and regenerative phases (Yin and Li, 2011; Yang et al., 2008; Ritzel et al., 2018).

Several studies have explored the relationship between specific immune cells, inflammatory markers, and their impact on susceptibility to IS (Sykes et al., 2021; Zhang et al., 2022; Dolati et al., 2018; Gu et al., 2019; Li et al., 2017; Kwan et al., 2013; Jefferis et al., 2013; Jefferis et al., 2011; Jefferis et al., 2009; Goncharov et al., 2024). However, the current literature examining immunosenescence in relation to IS risk is fragmented, limiting its translation into a unified understanding for clinical application. This narrative systematic review thus aims to pool current evidence on the association of senescent-associated immune cells and inflammatory markers with the risk of IS and outcomes after stroke. Unraveling these connections may give insights into potential clinical preventive tools, diagnostics and interventions that can bolster immune function in older adults and mitigate the impact of immunosenescence on the risk and outcomes of IS.

## Methods

### Search strategy and selection criteria

A systematic review was conducted to identify studies that investigated the relationship between immunosenescence and IS. The literature search was conducted on PubMed and Embase to identify relevant articles from the beginning of the database to 10 August 2024. Cohort studies that evaluated immunosenescence in IS patients aged 60 and above were included. Younger individuals–who are more likely to have autoimmune conditions that could confound the effects of immunosenescence–were not the focus of this review. The titles and abstracts of studies identified by this search strategy were screened for relevance and eligibility for this review. Only full-length research papers were considered for inclusion. They were required to include an analysis studying the association between the immunosenescence of at least one immune marker and either the risk or outcome of IS. Only studies including human participants and written in English were included. Research without original data, letter to editors, duplicate articles, congress abstracts and reviews were excluded. Articles reporting findings from the same study sample were included if they provided additional study findings that were not previously reported.

### Data extraction

The following indices were extracted from each article: first author, year of publication, study design, sample size, sample characteristics (age and gender), control size, control age, type of immune cells studied and results. Immune cells were classified as being measures of adaptive immune response and innate immune response.

### Selection of immune parameters

**Figure 1:**
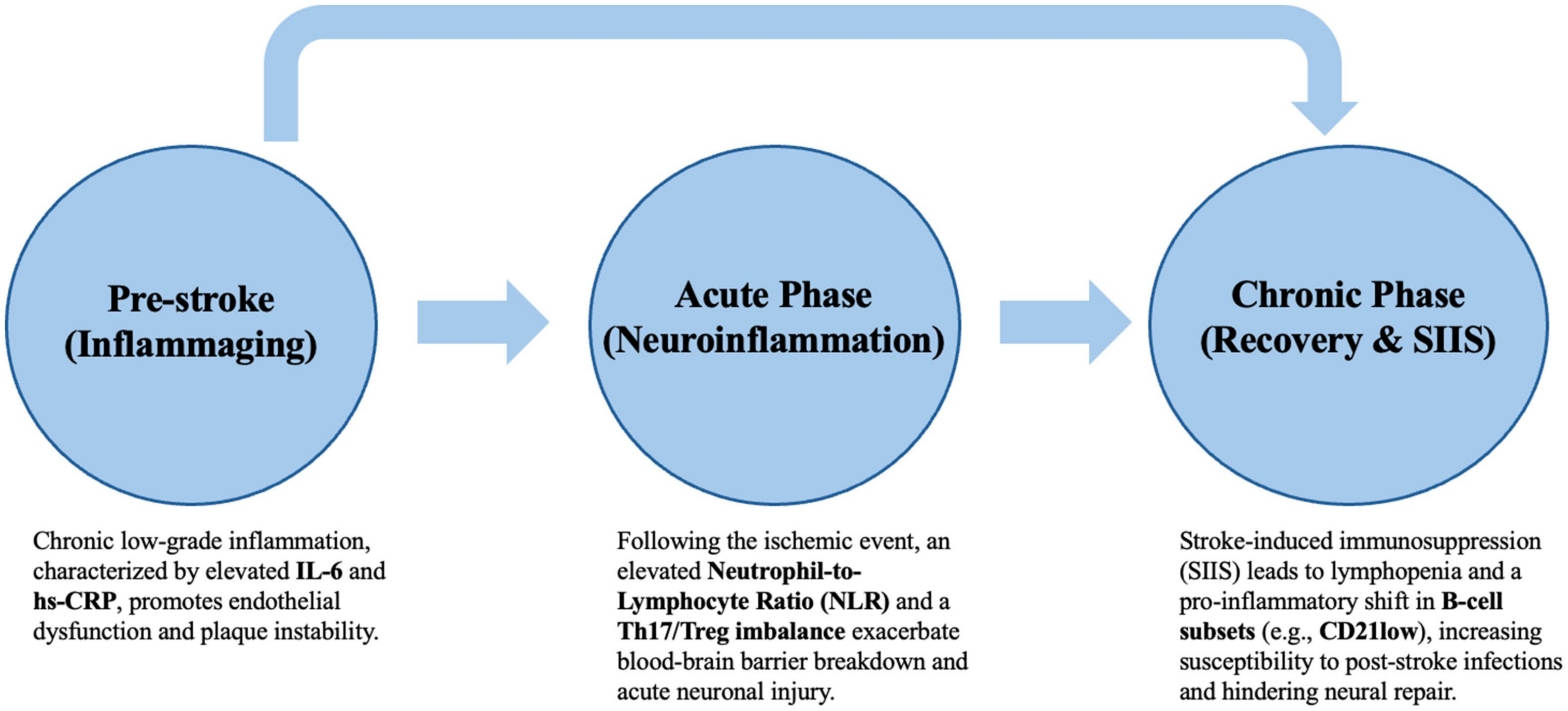
Hypothesized role of immunosenescence across stages of ischemic stroke. This schematic illustrates our proposed contribution of immune aging to stroke susceptibility and recovery. In the pre-stroke phase, 1 low-grade inflammation (“inflammaging”), characterized by elevated inflammatory markers such as IL-6 and hs-CRP, may increase stroke susceptibility through endothelial dysfunction and plaque instability. During the acute phase, stroke-triggered neuroinflammation is associated with innate immune activation, including an increased neutrophil-to-lymphocyte ratio (NLR) and Th17/Treg imbalance, which may exacerbate blood–brain barrier disruption and neuronal injury. In the chronic phase, stroke-induced immunosuppression (SIIS) can lead to lymphopenia and shifts toward pro-inflammatory B-cell subsets (e.g., CD21^low^), heightening infection risk and impairing recovery. Together, these processes highlight immunosenescence as a potential determinant of stroke risk and outcomes across the stroke trajectory.

The selection of specific immune markers in this review, notably IL-6, the Th17/Treg ratio, and the Neutrophil-to-Lymphocyte Ratio (NLR), was driven by their roles as representative pillars of the immunosenescence spectrum. We focused on IL-6 and hs-CRP as the canonical markers of ‘inflammaging’, the chronic, low-grade systemic inflammation that becomes pervasive with age. Both markers have been widely used in research and clinical settings, respectively, (Zhang et al., 2022; Gu et al., 2019; Li et al., 2017; Kwan et al., 2013; Jefferis et al., 2013; Jefferis et al., 2011; Jefferis et al., 2009). To address the adaptive side of immune aging, the Th17/Treg imbalance was selected to illustrate the shift toward a pro-inflammatory state and the concurrent loss of homeostatic control across various diseases (Dolati et al., 2018; Li et al., 2017; Goncharov et al., 2024). Finally, the NLR was included as a composite marker that reflects the tension between acute innate activation and adaptive exhaustion following an ischemic event. This marker has been described in large cohort studies and were found to be related to adverse clinical outcomes (Qiu et al., 2023). These relationships are visually synthesized in Figure 1.

## Results

**Figure 2:**
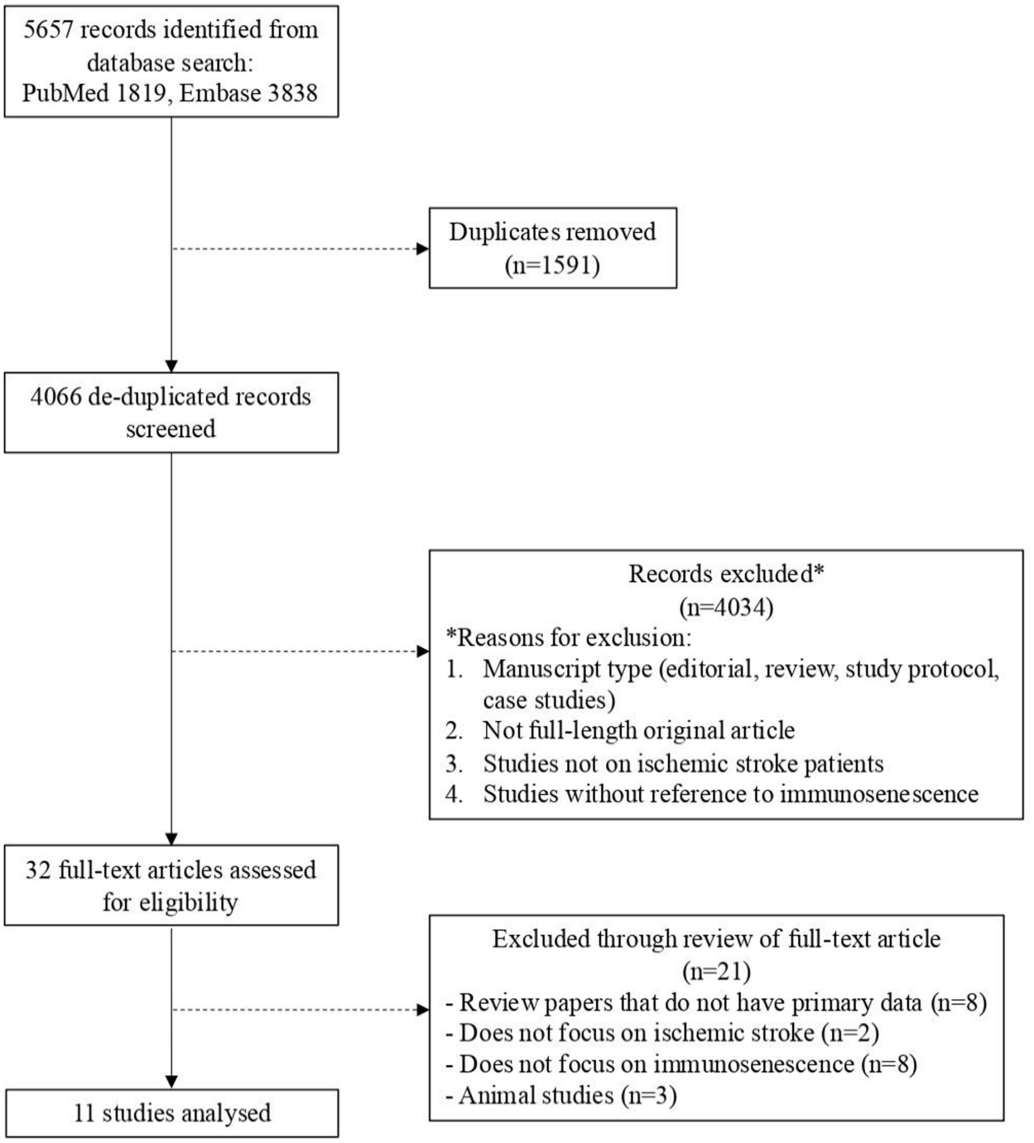
PRISMA flowchart depicting the study selection process. Full arrows indicate included articles, while dotted arrows represent excluded articles.

The initial search strategy identified 4,066 studies after removal of duplicated reports. After screening titles and abstracts, 4,034 studies were excluded based on the following criteria: non-original articles, studies not on IS patients, and those without reference to immunosenescence. The full texts of the remaining 32 articles were then assessed for eligibility, resulting in the exclusion of 21 articles. Ultimately, 11 articles were included in this systematic review (Sykes et al., 2021; Zhang et al., 2022; Dolati et al., 2018; Gu et al., 2019; Li et al., 2017; Kwan et al., 2013; Jefferis et al., 2013; Jefferis et al., 2011; Jefferis et al., 2009; Goncharov et al., 2024). The eligibility screening process, including reasons for exclusion, is detailed in the PRISMA flowchart (Figure 2).

The studies included were cohort studies (*n* = 8) that investigated the association between immune cell levels and the risk of IS (Sykes et al., 2021; Zhang et al., 2022; Dolati et al., 2018; Li et al., 2017; Jefferis et al., 2013; Jefferis et al., 2011; Jefferis et al., 2009; Goncharov et al., 2024). Two other studies examined post-stroke outcomes in stroke patients (Kwan et al., 2013; Qiu et al., 2023). Kwan et al. (2013) studied stroke-associated infections and mortality within the first 2 years after the stroke. Qiu et al. (2023) studied the prognostic value of the NLR in patients aged 80 years and older with acute IS. Another study investigated the association between elevated circulating inflammatory biomarkers and the presence of infarcts and microbleeds in neuroimaging (Gu et al., 2019). The principal characteristics of these studies are summarized in Table 1 and the significance of the results are detailed in Tables 2, 3.

**Table 1:** This table presents key characteristics of the included studies, including author and publication year, study design, and sample demographics.

Author	Year	Study design	Sample size	Sample Age, range	Sample Age, mean (SD)	Gender (male/female)	Control size	Control Age, mean (SD)	Race and ethnicity	Immune cells /markers measured	Main effect on IS studied	Source reference (DOI/PMID)
Dolati	2018	Patient vs. control	30	62–74	67.3 ± 8.9	19/11	30	NR	NR	Th17 Treg RORγt microRNA-326 microRNA-106b microRNA-25 IL-17A TGF-beta	Risk of IS	DOI: 10.1007/s10072-018-3250-4
Sykes	2021	Patient vs. control	94	37.8–89.6	65.9 ± 12.5	50/50	79	63.8 ± 12.6	Caucasian: 69.1%, African-American: 11.7%, Asian: 5.3%, Hispanic: 3.2%, Others: 10.6%	CR2 (or CD21) CD79B CD27 CCR7 RAS-GRP3 NT5E (or CD73) B-cell IL-7 T-helper cell	Risk of IS	DOI: 10.1161/strokeaha.120.032040
Jefferis	2009	Patient vs. control	299	60–79	71.29 ± 5.32	191/108	587	71.35 ± 5.24	NR	TNF-alpha CRP IL-6 Fibrinogen White cell count	Risk of IS	DOI: 10.1016/j.atherosclerosis.2008.12.001
Jefferis	2011	Patient vs. control	304	60–79	71.36 ± 5.32	194/110	596	71.39 ± 5.26	NR	sCD40L CRP IL-6 Fibrinogen White cell count	Risk of IS	DOI: 10.1111/j.1538-7836.2011.04415.x
Jefferis	2013	Patient vs. control	300	60–79	71.32 ± 5.33	191/109	590	71.34 ± 5.26	NR	IL-18 CRP IL-6 Fibrinogen White cell count	Risk of IS	DOI: 10.1016/j.cyto.2012.10.010
Li	2017	Patient vs. control	36	–	73.5 ± 6.9	21/15	≥65: 34 45–64: 33 ≤44: 30	33.9 ± 6.9 52.8 ± 7.1 70.4 ± 5.6	NR	Th17 IL-17 IL-6 hs-CRP RORγt	Risk of IS	PMCID: PMC5714799
Zhang	2022	Different-aged patients (no control)	41 A (<55): 13 B (55–65): 18C (>65): 10	18–80	A: 43.85 ± 7.57 B: 59.17 ± 3.11 C: 68.1 ± 2.42	A: 10/3 B: 13/5C: 7/3	NR	NR	NR	White cell count Neutrophil count hs-CRP CD56 + CD16dim NK cells CD16 − CD14 + monocytes CD16 + CD14 + monocytes CD3 + T-cells CD3 + CD4 + T-cells CD19 + B-cells	Risk of IS	DOI: 10.3389/fneur.2022.887526
Gu	2018	MRI findings	IL6: 357	-	72.56 (5.68)	120/237	NR	NR	Caucasian: 31%, African-American: 31.3%, Hispanic: 36.2%, Others: 1.8%	IL6 CRP ACT	Risk of IS	DOI: 10.1212/NXI.0000000000000521
Goncharov	2024	Patient vs. control	24	60–75	67.5	10/14	23	65	NR	DP Th17 cells Th17-like cells NK cells B-cells	Risk of IS	DOI: 10.3390/ijms25031888
Kwan	2013	Patient vs. control	45 (with SAI)	–	75.6 ± 10.7	21/24	37 (without SAI)	73.0 ± 10.6	NR	IL-6 TNF-alpha IL-1b	Outcomes after IS (SAI)	DOI: 10.1016/j.exger.2013.07.003
Qiu	2023	Patient (no control)	476	≥80	84	248/228	NR	NR	NR	NLR	Outcomes after IS (90-day prognosis)	DOI: 10.1272/jnms.JNMS.2023_90-110

NR = not reported.

**Table 2:** Comparison of inflammatory markers and their reported significance in ischemic stroke.

Immune cells/markers	*p*-value											Total sample size (N)*	Studies with significant association	Relative consistency	
	Dolati et al. (2018) 30	Sykes et al. (2021) 173	Jefferis et al. (2009) 299	Jefferis et al. (2011) 304	Jefferis et al. (2013) 300	Li et al. (2017) 36	Zhang et al. (2022) 41	Gu et al. (2019)	Goncharov et al. (2024)	Kwan et al. (2013) 82	Qiu et al. (2023) 476				
TNF-alpha												381	0/2	Low	
CRP								NR				980	1/5	Low	
IL-6								NR				1,021	5/5	High	
Fibrinogen												903	0/3	Low	
White cell count												944	3/3	High	
Neutrophil count															
NLR															
sCD40L															
IL-1-beta															
IL-7															
IL-17															
IL-18															
CR2 (CD21)															
CD79B															
CD27															
CCR7															
RAS-GRP3															Not tested
NT5E (CD73)															*p* > 0.05
Alpha 1-antichymotrypsin								NR							*p* < 0.05
microRNA-25															*p* < 0.01

**Table 3:** Comparison of immune cell populations and their reported significance in ischemic stroke.

	*p*-value											
	Dolati et al. (2018)	Sykes et al. (2021)	Jefferis et al. (2009)	Jefferis et al. (2011)	Jefferis et al. (2013)	Li et al. (2017)	Zhang et al. (2022)	Gu et al. (2019)	Goncharov et al. (2024)	Kwan et al. (2013)	Qiu et al. (2023)	
Innate immune cells												
CD56 + CD16dim NK cells												
CD16 − CD14 + monocytes												
CD16 + CD14 + monocytes												
Adaptive immune cells												
Th17	Post-stroke day 1					Memory						
	Post-stroke day 5					Naive						
	Post-stroke day 10											
Treg	Post-stroke day 1											
	Post-stroke day 5											
	Post-stroke day 10											
FOXp3 expression	Post-stroke day 1											
	Post-stroke day 5											
	Post-stroke day 10											
RORγt expression	Post-stroke day 1											
	Post-stroke day 5											
	Post-stroke day 10											
T-helper cell differentiation												Not tested
CD3 + T-cells												*p* > 0.05
CD3 + CD4 + T-cells												*p* < 0.05
CD19 + B-cells												*p* < 0.01

### Innate immune system

#### Inflammatory markers

Interleukin (IL)-6: IL-6 was associated with worse stroke outcomes in several studies. Jefferis et al. (2009, 2011, 2013) found that IL-6 significantly increases the risk of developing stroke. Gu et al. (2019) and Kwan et al. (2013) observed that CRP and IL-6 levels were higher in patients with infarcts and Stroke-Associated Infections (SAI). The study by Kwan et al. (2013) highlighted that patients who subsequently developed SAI exhibited elevated levels of IL-6 and other inflammatory markers, such as TNF-alpha, during the first 72 h of admission for stroke. Specifically, patients with confirmed pneumonia had significantly higher IL-6 levels and worse stroke severity metrics, including increased National Institutes of Health Stroke Scale (NIHSS) and Modified Rankin Scale (mRS) scores. IL-6 was found to be a strong independent predictor of SAI, even when adjusting for other risk factors and inflammatory biomarkers. IL-6 levels also predicted long-term mortality, demonstrating a significant association with decreased survival rates at 2 years post-stroke. In the study by Gu et al. (2019), elevated levels of IL-6 were significantly associated with an increased likelihood of having baseline infarcts, as assessed through MRI scans. Each standard deviation increase in IL-6 was linked to 30% higher odds of presenting with infarcts at the initial MRI scan. In contrast, while higher levels of CRP and activated complement component (ACT) showed some association with cerebrovascular damage, only IL-6 remained significantly linked to baseline infarcts after adjusting for various factors such as body mass index, smoking status, and depression. This age-related increase in IL-6 is also deeply intertwined with cardiovascular frailty. In older adults, the synergy between a high burden of senescent cells and frailty - a state of reduced physiological reserve - creates a low threshold for ischemic events and a limited capacity to survive the metabolic and inflammatory stress of a stroke (Winovich et al., 2017).

Interleukin IL-17: IL-17 is produced by multiple T cell subsets, including T helper 17 (Th17) cells, gamma delta (γδ) T cells, alpha beta (αβ) T cells, and natural killer T cells (Navarro-Compan et al., 2023). Li et al. (2017) noted that IL-17 levels were significantly elevated in stroke patients. Sykes et al. (2021) found that changes in IL-17 signaling and reductions in markers such as cluster of differentiation (CD)27 and CCR7 reflect a decline in T-cell functionality, with older patients exhibiting a higher proportion of exhausted IL-17R^low^ CD8 + T-cells.

Interleukin IL-18: Jefferis et al. (2013) revealed that although IL-18 levels were associated with several inflammatory and haemostatic markers (such as CRP, IL-6, fibrinogen, and white blood cell count), as well as established cardiovascular risk factors like blood pressure and triglycerides, they did not show a strong independent association with stroke risk. Specifically, geometric mean IL-18 levels were similar between stroke cases and controls, and there was no significant association between IL-18 levels and stroke outcomes. Although IL-18 levels were positively correlated with diabetes and negatively associated with high-density lipoprotein cholesterol, these associations did not translate into a significant predictive value for stroke risk.

High-sensitivity CRP (hs-CRP): Li et al. (2017) reported significantly elevated levels of hs-CRP in elderly patients with acute IS compared to healthy controls. In contrast, Zhang et al. (2022) and Jefferis et al. (2009, 2011, 2013) found no statistically significant differences in hs-CRP levels among different age groups in their studies.

Soluble CD40 ligand (sCD40L): The association of sCD40L with stroke risk was evaluated by Jefferis et al. (2011). The study found no significant difference in sCD40L levels between stroke cases and controls. Although sCD40L levels were associated with smoking and other inflammatory markers such as IL-6 and white blood cell count, there was no robust evidence linking sCD40L levels to stroke risk across the study population. Notably, a gender-specific analysis revealed a potential association in men, with higher sCD40L levels correlating with increased stroke risk, but this finding was not significant for women.

CD31, CD34, CD133, CD147: Endothelial markers found in plasma were studied by Goncharov et al. (2024), comparing between a population with acute IS and a control cohort. There was an increase in endothelial-derived microparticles and platelet-derived microvesicles, which were implicated in inflammation and thrombosis. The acute IS group showed elevated levels of CD31 and CD147, indicating enhanced endothelial activation and dysfunction. The levels of CD34 and CD133, markers associated with endothelial progenitor cells, were notably altered, suggesting disruptions in endothelial repair mechanisms.

TNF-alpha: Jefferis et al. (2009) found that TNF-alpha levels were slightly elevated in stroke cases compared to controls, but this difference was not statistically significant. Despite these elevated levels, TNF-alpha did not show a significant association with stroke risk in multivariate analyses, with an odds ratio of 1.12 for the highest versus lowest TNF-alpha tertile and no significant correlation with continuous TNF-alpha levels. Additionally, there were no interactions between TNF-alpha and variables such as gender or age in relation to stroke risk.

#### White cell counts

Qiu et al. (2023) found that higher NLR, indicative of increased systemic inflammation, was independently associated with poorer 90-day outcomes in patients aged 80 and above. The research highlighted that neutrophils, as part of the acute inflammatory response, accumulate in the ischemic brain tissue and contribute to the inflammation process. It did not differentiate between specific subtypes of neutrophils. Conversely, there is a relative decrease in lymphocytes due to post-stroke immunosuppression. The study observed that elderly patients exhibited a higher median NLR compared to younger individuals, suggesting more severe inflammation in this age group. The observed lymphopenia in older patients is a hallmark of stroke-induced immunosuppression (SIIS). SIIS is a systemic phenomenon where the central nervous system, via the autonomic nervous system and the HPA axis, triggers a rapid depletion of peripheral lymphocytes to prevent autoimmunity against brain antigens (Westendorp et al., 2022). In the aging population, SIIS is particularly dangerous as it is superimposed on a pre-existing immunosenescent state, further increasing the risk of life-threatening post-stroke infections.

#### Natural killer (NK) cells

Goncharov et al. (2024) observed a slight increase in NK cell proportion from 11.31% in healthy controls to 13.64% in patients with acute IS, suggesting a potential role of NK cells in stroke pathophysiology. Zhang et al. (2022) found no significant difference in NK cells between IS patients and healthy controls. However, the authors did identify a positive correlation between CD56 + CD16^dim^ NK cells and hs-CRP levels (*p* = 0.011), indicating an association between certain NK cell subsets and inflammatory markers. They also found that the proportion of CD56 + CD16^dim^ NK cells was lowest in the 55–65 age group compared to older and younger groups, though this difference was not statistically significant.

### Adaptive immune system

#### T cell subsets

Th17, Regulatory T (Treg): Dolati et al. (2018) found an increase in the proportion of Th17 cells from 2.2% in healthy controls to 4.68 and 3.25% in patients one- and five-days post-stroke, respectively. The levels return to near baseline by day ten. They also found that the frequency of Treg cells, which are crucial for immune suppression and maintaining homeostasis, was significantly reduced at one- and five-days post-stroke, from 10.17% in controls to 6.83 and 8.47% in stroke patients. This reduction persisted at day ten, although not significantly different from controls. They observed an increased levels of RORγt, a transcription factor driving Th17 differentiation, and decreased levels of FoxP3, a marker of Treg cells, further indicating an imbalance between pro-inflammatory Th17 and anti-inflammatory Treg cells. Additionally, elevated levels of IL-17A and decreased levels of TGF-*β*, which are associated with Th17 and Treg cell function respectively, were found in stroke patients. Of the microRNAs (miR) related to Th17 and Treg cell differentiation, they found an upregulation of miR-326, miR-106b, and miR-25 in stroke patients, which suggests potential biomarkers and therapeutic targets. Goncharov et al. (2024) also observed an increase in Th17-like cells. Li et al. (2017) found that elderly IS patients had significantly higher frequencies of Th17 cells (CD4 + IL-17+) compared to healthy age-matched controls, with Th17 levels also elevated in healthy elderly individuals relative to younger cohorts. The study also found that endothelial cell senescence, indicated by elevated as endothelin-1 (ET-1) and vascular cell adhesion molecule-1 (VCAM-1) levels and decreased endothelial nitric oxide synthase levels, was pronounced in elderly acute IS patients. Notably, IL-17A, a key cytokine produced by Th17 cells, was shown to induce endothelial cell senescence *in vitro*, further linking Th17 cells to the pathogenesis of acute IS.

CD3+, CD4+: Zhang et al. (2022) revealed a significant decrease in CD3 + and CD3 + CD4 + T-cells with age, suggesting reduced T-cell activity in older patients. Conversely, CD3 + CD4 − CD8 − T-cells and CD3 + CD8 + T-cells increased with age. Monocyte and B-cell proportions did not show significant age-related variations.

In summary, Li et al. (2017) and Dolati et al. (2018) both highlight the critical role that the balance between Th17 and Treg cells plays in the pathogenesis of IS, pointing to the Th17/IL-17 pathway as a promising target for therapy. Goncharov et al. (2024) expanded on this by showing specific increases in double positive (DP) Th17 cells (CXCR3 + CCR4+) in acute IS patients, though these changes were not uniquely linked to stroke development. On the other hand, Sykes et al. (2021) examined how aging impacts T-cell functionality more broadly, without focusing specifically on the Th17/Treg balance. Analyses of individual immune cell subsets reveal small changes that individually have a minor impact on the associated risk profiles. However, from these reports we can infer that multiple, diverse immune cell types are clearly implicated and collectively these may have a significant cumulative effect on IS risk.

#### B cell subsets

Sykes et al. (2021) found that older patients showed a marked decrease in the expression of key genes involved in B-cell and T-cell function. For instance, genes like CR2 (CD21), MS4A1 (CD20), and CD79, important for B-cell activation and antibody production, diminish with age. On the other hand, Zhang et al. (2022) found that the distribution patterns of monocytes and B cells did not vary significantly across different age groups.

## Discussion

Understanding how immune aging shapes stroke susceptibility and recovery is increasingly important in the context of global population aging. While multiple studies have examined individual immune cell subsets and inflammatory markers, their collective implications for immunosenescence and IS remain fragmented. This review brings together current evidence to clarify these relationships, identify key gaps, and support the development of clinically actionable strategies to improve outcomes in older adults.

### Association of immune cell types on ischemic stroke risk and outcomes

#### Innate immune system

##### Inflammatory markers

The role of proinflammatory cytokines such as IL-6, IL-17, TNF-alpha, and CRP in stroke pathophysiology has been a significant area of research, underscoring their potential as biomarkers for IS risk and outcomes.

IL-6 is one of the most consistently reported biomarkers across multiple studies. The work of Jefferis et al. (2009, 2011, 2013) and Gu et al. (2019) highlights IL-6 as a relatively stable inflammatory marker that correlates with increased stroke risk (Sun et al., 2023). IL-6 plays a key role in promoting both local and systemic inflammatory responses, which can lead to endothelial dysfunction, vascular remodeling, and atherogenesis—pathophysiological processes that are integral to stroke development (Hou et al., 2008; Su et al., 2021; Katkenov et al., 2024). With increased age, IL-6 levels rise due to chronic low-grade inflammation, often termed “inflammaging” (Ferrucci and Fabbri, 2018). Both the heightened inflammatory state and the declining ability of the aged immune system to resolve inflammation effectively increase susceptibility to stroke (Papadopoulos et al., 2022; Zhu et al., 2022; Jenny et al., 2019). IL-6 has also been identified as an independent predictor of post-stroke outcomes, such as SAI and mortality (Kwan et al., 2013; Faura et al., 2021; Yang et al., 2020). While IL-6 has shown consistent associations with stroke risk, other markers such as TNF-alpha and IL-18 have yielded less conclusive results. Jefferis et al. (2009, 2011, 2013) found no significant association between these cytokines and stroke risk. TNF-alpha is known to promote inflammation by activating neutrophils and increasing the expression of adhesion molecules on endothelial cells, thereby facilitating leukocyte infiltration into the brain parenchyma during ischemic events (Chandrasekharan et al., 2007). This suggests that not all proinflammatory cytokines are equally involved in stroke pathophysiology (Zhang et al., 2021).

hs-CRP has also been extensively studied as a marker of systemic inflammation and its role in IS. CRP is an acute-phase protein produced by the liver in response to IL-6, and its levels increase in response to systemic inflammation (Sproston and Ashworth, 2018). Elevated CRP levels reflect an ongoing inflammatory process that contributes to endothelial dysfunction, plaque instability, thereby increasing likelihood of cerebrovascular events like IS (Banait et al., 2022). Li et al. (2017) demonstrated that elderly patients with acute IS had significantly elevated hs-CRP levels compared to healthy controls, supporting the notion that CRP is a sensitive marker of vascular inflammation (Banait et al., 2022). Despite these findings, the utility of hs-CRP as a universal marker of stroke risk, particularly in older adults, remains debated. Zhang et al. (2022) and Jefferis et al. (2009, 2011, 2013) found no statistically significant differences in hs-CRP levels among different age groups in their studies. This discrepancy could be due to variations in study populations, comorbidities, or even differences in the timing of CRP measurement relative to stroke onset. While hs-CRP is clearly elevated in acute inflammatory states such as IS, its ability to reflect the chronic, subtle changes in immune function associated with aging and immunosenescence may be limited (Zhang et al., 2021).

Sykes et al. (2021) revealed that aging is associated with reduced signaling via IL-7, a cytokine crucial for T-cell development and maintenance. As individuals age, the diminished effectiveness of IL-7 signaling leads to a higher proportion of IL-7 receptor^low^ CD8 + T-cells (Kim et al., 2006; Ucar et al., 2017; Aspinall et al., 2014). These cells exhibit the characteristics of terminally differentiated effector memory T (T-EM) cells, such as reduced proliferative and cytokine-producing capabilities (Fülöp et al., 2013). This shift in T-cell populations results in a decreased ability to mount an effective immune response to new infections and injuries, including IS. T-EM cells, which are more prevalent in older individuals, may contribute to a pro-inflammatory environment, leading to increased tissue damage and poorer outcomes post-stroke (Lazuardi et al., 2005). Additionally, studies have shown that the aged immune system’s increased proinflammatory state may not only hinder recovery but also elevate the risk of secondary infections, which are common complications in stroke patients (Yu et al., 2024).

##### White cell counts

Qiu et al. (2023) highlighted that a high NLR is a significant independent risk factor for acute IS in individuals aged 80 and older, with its prognostic value extending to outcomes 90 days post-stroke. This finding supports the idea that NLR serves as a composite marker of systemic inflammation, reflecting both an increase in neutrophils (neutrophilia) and a decrease in lymphocytes (lymphopenia). These changes suggest an activated innate immune response alongside a weakened adaptive immune function. These shifts are especially pertinent, as inflammaging can predispose older adults to poorer outcomes following an ischemic event (Teissier et al., 2022). The elevated NLR in this age group may indicate a heightened inflammatory response, which can also contribute to the development of stroke by promoting clot formation, disrupting blood vessel function, and worsening neuronal injury (Quan et al., 2021; Li et al., 2022; Sharath et al., 2022; Li et al., 2021).

The studies by Jefferis et al. (2009, 2011, 2013) consistently found higher white blood cell counts, particularly driven by an increase in neutrophils, in IS patients. This underscores the acute inflammatory response that characterizes ischemic events. Neutrophils, as frontline defenders in the innate immune system, are quickly mobilized to sites of injury where they release enzymes, reactive oxygen species, and pro-inflammatory cytokines. These actions contribute to the breakdown of the blood–brain barrier, worsening neuronal damage, and promoting thromboinflammation—a key factor in the progression of IS (Rosales et al., 2017; Fine et al., 2020; Simats and Liesz, 2022). However, Zhang et al. (2022) reported no statistically significant differences in white blood cell or neutrophil counts across different age groups and found no direct correlation between neutrophil counts and specific immune cell subsets. This suggests that neutrophil count alone may not be a sufficiently sensitive or specific marker to fully capture the range of immune system changes associated with IS, particularly when viewed in isolation. For instance, the functional state of neutrophils—whether they are promoting or resolving inflammation—might be more relevant than their numbers (Loh and Vermeren, 2022). Additionally, the interaction between neutrophils and other immune cells, such as lymphocytes, monocytes, and brain-resident microglia, plays a crucial role in shaping the overall inflammatory response.

##### NK cells

NK cells are a crucial component of the innate immune system due to their ability to target and eliminate virus-infected cells and malignancies without prior sensitization. The slight increase in NK cell proportion observed by Goncharov et al. (2024) in acute IS patients may suggest an innate immune response aimed at mitigating neuronal damage or controlling the systemic inflammatory response triggered by cerebral ischemia (Ning et al., 2023). The elevated proportion of NK cells in IS patients, compared to healthy controls, could be indicative of an acute immune activation state, where NK cells are recruited to the site of injury or mobilized into circulation as part of the body’s initial defense mechanism (Lodoen and Lanier, 2006). Zhang et al. (2022) showed no significant difference in overall NK cell levels between IS patients and healthy controls. However, they observed a positive correlation between CD56 + CD16 ^dim^ NK cell subsets and hs-CRP levels. An increase in CD56 + CD16 ^dim^ NK cells are associated with a reduced proliferative capacity and altered receptor expression (Gayoso et al., 2011; Sanchez-Correa et al., 2016). CD56 + CD16^dim^ NK cells are typically associated with regulatory functions and a higher cytotoxic potential compared to their CD56 + CD16^bright^ counterparts (Kamath et al., 2015; Shereck et al., 2019). Hence, an increased CD56 + CD16^dim^ NK cells and elevated hs-CRP levels in stroke patients may suggest that these cells are actively engaged in the immune response to cerebral ischemia, indicating an attempt by the immune system to control tissue damage and inflammation through direct cytolytic activity (Paul and Lal, 2017). hs-CRP is a well-established marker of inflammation, and its association with CD56 + CD16^dim^ NK cells suggests that these cells may contribute to or be a consequence of the inflammatory state in stroke patients. However, this increased activity could also contribute to secondary damage, exacerbating the inflammatory response and leading to worse outcomes (Ma et al., 2021). In Zhang et al. (2022), the greatest proportion of CD56 + CD16^dim^ NK cells has been observed in the age group with older adults aged 65 and above, leaving individuals in this age group more susceptible to severe inflammatory responses and poorer outcomes post-stroke.

#### Adaptive immune system

##### T cell subsets

Zhang et al. (2022) found that CD3 + and CD4 + T-cells is lower in proportion in older age groups compared to younger ones. This finding is consistent with the existing knowledge that aging is associated with a decline in naive T-cells and an increase in memory and senescent T-cells (Zhang et al., 2022). While lower levels of CD3 + T-cells might suggest a reduced inflammatory response, the specific nature of the CD4 + T-cell subsets is critical in interpreting these results. CD4 + T-cells encompass a variety of subtypes with both pro-inflammatory (e.g., Th1, Th17) and anti-inflammatory (e.g., Treg) roles, and their balance is crucial in the immune response to stroke (Zhang et al., 2022). The study by Zhang et al. (2022) did not provide a detailed analysis of these subtypes, making it difficult to draw definitive conclusions about their role in post-stroke outcomes. Moreover, the results regarding CD3+ and CD4+ T-cells were not statistically significant, However, other studies have demonstrated that a shift toward Th2 immunity is associated with a reduced risk of myocardial infarction and stroke. For instance, Engelbertsen et al. (2013) found that high numbers of Th2 cells and the release of the Th2 cytokine interleukin-4 were independently associated with a reduced risk of cardiovascular events, including stroke (Engelbertsen et al., 2013). Additionally, Wong et al. (2017) reported prolonged activation of invariant NK T cells and Th2-skewed immunity in stroke patients, suggesting that Th2 responses may play a protective role in post-stroke outcomes (Wong et al., 2017). These findings highlight the potential significance of Th2 immunity in reducing the risk of adverse cardiovascular events.

Th-17 and Treg cells were extensively studied and were found to play a significant role in the development and progression of IS, particularly through their involvement in promoting inflammation and endothelial cell senescence. Findings by Li et al. (2017), Dolati et al. (2018) and Goncharov et al. (2024) on Th17 cells are consistent with existing literature, highlighting the Th17/IL-17 pathway and IL-17A as critical factors in IS development (Lu et al., 2022; Shu et al., 2024; Hu et al., 2014). The results also imply that Th17/Treg dysregulation, evidenced by increased Th17 and reduced Treg cells, plays a role in IS pathogenesis. In aged and immunosenescent individuals, Th17 cells to Tregs ratio appeared to be higher and thus promote a basal proinflammatory state, whereas with stimulation, this ratio decreased in aged individuals (Schmitt et al., 2013). Presumably, this is because it is difficult to further stimulate an already activated system, leading to a reduced response despite additional stimulation. It is also not shown through these studies the effect of T17/Treg cell ratio on the outcomes post-IS, but another review suggested that increased Treg cell numbers may be associated with poorer prognosis of IS (Chen et al., 2024). On one hand, in animal studies, Treg cells have demonstrated an ability to enhance long-term recovery after a stroke by boosting the repair functions of microglia and supporting the regeneration of oligodendrocytes and the repair of damaged white matter (Shi et al., 2021). On the other hand, Treg cell expression have been found to worsen functional outcomes and that lower levels of Treg improved neurological function post stroke (Kleinschnitz et al., 2013; Schuhmann et al., 2015). Goncharov et al. (2024) also revealed that biomarkers showing significant changes from healthy controls included a relative increase in Th17-like cells and both a relative and absolute increase in DP Th17 cells. However, the study noted that these changes are not specific to the development of acute IS.

##### B cell subsets

B cells play a key role in regulating inflammation and presenting antigens (Rastogi et al., 2022; de Gruijter et al., 2022). Sykes et al. (2021) found a reduction in several markers critical for B-cell receptor (BCR) signaling—such as CR2 (CD21), CD27, CCR7, and NT5E (CD73)—indicating a decline in B-cell activation and antibody production in older adults. The decrease in CR2 levels is particularly important, as CR2 is involved in activating B cells in response to antigens and regulating the immune response to prevent excessive inflammation (Noris and Remuzzi, 2013; Nielsen et al., 2000). CR2, also known as CD21, interacts with complement fragments such as C3d, which are attached to antigens, thereby lowering the threshold for B cell activation and enhancing antibody production (Erdei et al., 2021; Kovacs et al., 2021). Reduced CR2 may impair these regulatory roles, leading to a shift toward more pro-inflammatory B cells. Sykes et al. observed an increase in the pro-inflammatory CD21^low^ B-cell subset, which lacks CR2 and is known for producing higher levels of inflammatory cytokines like TNF-alpha, IL-6, and IFN-gamma. This pro-inflammatory shift can worsen stroke outcomes in elderly patients. These findings align with broader research showing that B-cell function declines with age. Aging reduces the output of new B cells from the bone marrow and diminishes the diversity and function of memory B cells, which are crucial for rapid responses to previously encountered pathogens (Frasca et al., 2020). However, it is important to consider that the observed reduction in CR2 expression in RNA from peripheral blood could be due to a lower number of B cells present rather than downregulated expression by the B cells themselves. B cells may also play a role in stroke recovery beyond antibody production. Regulatory B cells (Bregs), for example, have anti-inflammatory properties that help control excessive immune responses (Ahsan et al., 2024). A reduction in these protective B-cell subsets with age could contribute to a more pronounced inflammatory state after a stroke, complicating recovery. Conversely, Zhang et al. (2022) reported that the distribution of monocytes and B cells did not vary significantly across age groups, suggesting a more complex picture of how aging affects the immune system. While the total number of B cells may not show significant change with age, their functionality and phenotype might be altered in the context of inflammation following a stroke.

#### Overall synthesis

While various markers were investigated, IL-6 emerges as the most robust ‘gold-standard’ candidate for both risk prediction and therapy in older adults. Our synthesis of 1,021 participants across five cohorts consistently links elevated IL-6 to increased stroke risk, baseline infarct volume, and post-stroke mortality. Unlike TNF-alpha or IL-18, which showed inconsistent independent associations, IL-6 remains a significant predictor even after adjusting for traditional cardiovascular risk factors, marking it as a primary target for translational research.

These markers serve distinct clinical roles. We propose IL-6 as a ‘dual-action’ marker: it functions as a predictive tool for post-stroke infection risk and a direct therapeutic target for mitigating ‘inflammaging’. Conversely, the Th17/Treg ratio and NLR act primarily as indicators of disease severity and prognostic tools for 90-day recovery. Priority should be given to markers with high consistency, specifically IL-6 and NLR, as they bridge the gap between systemic inflammation and clinical mortality. Routine measurement of these markers in geriatric practice could refine stroke prevention. For instance, elevated baseline IL-6 may identify ‘high-risk’ individuals who would benefit from aggressive vascular management, allowing for a shift toward early intervention before an ischemic event occurs.

## Limitations

Our systematic review focused exclusively on human studies. On assessing the quality of studies analyzed, the sample sizes of Dolati et al. (2018), Li et al. (2017), and Goncharov et al. (2024) are 30, 36 and 24 respectively, which are relatively small and may limit the reliability of their results. Ethnicity data was not consistently reported across all studies, except in Gu et al. (2019) and Sykes et al. (2021). This is a significant limitation, as racial differences in immune function may influence the findings. There is also a lack of controls in the studies conducted by Zhang et al. (2022), Gu et al. (2019), and Qiu et al. (2023). In addition, the large heterogeneity in study design in the papers above may limit the effectiveness in comparing the various immune cells and inflammatory markers measured. Many studies assessed cytokine levels and immune markers at a single time point, often immediately post-stroke, without examining baseline immune function. This makes it difficult to determine whether immune alterations are predisposing factors for stroke or merely a consequence of the event. Future studies should incorporate baseline immune profiling to better assess causality. Additionally, a more comprehensive evaluation of immune function, including antigenic stimulation assays such as toll-like receptor stimulation to assess cytokine responses of immune cells, would provide deeper insights into immune dysregulation in stroke. Future research should also explore the interactions between the peripheral immune system and CNS endothelial cells, as these crosstalk mechanisms are likely to play a key role in stroke pathophysiology. Due to significant heterogeneity of the markers studied, our systematic review is narrative as meta-analysis could not be performed. These suggest that more research done in a standardized approach would be required to further confirm the findings presented above.

## Conclusion

In summary, a pro-inflammatory environment is a key feature in the development and progression of stroke. Of note, inflammatory markers associated with worse outcomes are IL-6, hs-CRP, IL-7, Th17 cells and the Th17/Treg ratio, and CD21^low^ B-cell. The evidence points to chronic inflammation, worsened by the aging immune system, as playing a major role in both making individuals more susceptible to stroke and affecting their recovery afterward. Our study is important in guiding future work on how immunosenescence affects the risk and outcomes of IS. Ultimately, this can lead to discovery of immune-related risk prediction tools for earlier intervention, prognostic and therapeutic targets that can bolster immune function in older adults with stroke and reduce consequent morbidity and mortality.

## Data Availability

The original contributions presented in the study are included in the article/supplementary material, further inquiries can be directed to the corresponding author/s.
